# Epigenetic Silencing in Friedreich Ataxia Is Associated with Depletion of CTCF (CCCTC-Binding Factor) and Antisense Transcription

**DOI:** 10.1371/journal.pone.0007914

**Published:** 2009-11-19

**Authors:** Irene De Biase, Yogesh K. Chutake, Paul M. Rindler, Sanjay I. Bidichandani

**Affiliations:** 1 Department of Biochemistry and Molecular Biology, University of Oklahoma Health Sciences Center, Oklahoma City, Oklahoma, United States of America; 2 Department of Pediatrics, University of Oklahoma Health Sciences Center, Oklahoma City, Oklahoma, United States of America; Ohio State University Medical Center, United States of America

## Abstract

**Background:**

Over 15 inherited diseases are caused by expansion of triplet-repeats. Friedreich ataxia (FRDA) patients are homozygous for an expanded GAA triplet-repeat sequence in intron 1 of the *FXN* gene. The expanded GAA triplet-repeat results in deficiency of *FXN* gene transcription, which is reversed via administration of histone deacetylase inhibitors indicating that transcriptional silencing is at least partially due to an epigenetic abnormality.

**Methodology/Principal Findings:**

We found a severe depletion of the chromatin insulator protein CTCF (CCCTC-binding factor) in the 5′UTR of the *FXN* gene in FRDA, and coincident heterochromatin formation involving the +1 nucleosome via enrichment of H3K9me3 and recruitment of heterochromatin protein 1. We identified FAST-1 (*FXN* Antisense Transcript – 1), a novel antisense transcript that overlaps the CTCF binding site in the 5′UTR, which was expressed at higher levels in FRDA. The reciprocal relationship of deficient *FXN* transcript and higher levels of FAST-1 seen in FRDA was reproduced in normal cells via knockdown of CTCF.

**Conclusions/Significance:**

CTCF depletion constitutes an epigenetic switch that results in increased antisense transcription, heterochromatin formation and transcriptional deficiency in FRDA. These findings provide a mechanistic basis for the transcriptional silencing of the *FXN* gene in FRDA, and broaden our understanding of disease pathogenesis in triplet-repeat diseases.

## Introduction

Friedreich ataxia (FRDA), the most common inherited ataxia, is an autosomal recessive disease characterized by progressive sensory ataxia, cardiomyopathy, diabetes, and premature death [1]. FRDA is most commonly caused by inheriting an expanded GAA triplet-repeat sequence in intron 1 of both copies of the *FXN* gene [2]. The size of the expanded repeat tract can range from 66–1700 triplets, which results in a deficiency of *FXN* gene transcription [3], [4]. This in turn causes a deficiency of the mitochondrial protein frataxin, which is essential for iron-sulfur cluster biogenesis, and thereby results in mitochondrial dysfunction [1].

Exactly how *FXN* transcriptional silencing is achieved in FRDA is not well understood, however recent evidence indicates that an epigenetic abnormality is an important underlying mechanism. In unrelated transgenic mouse experiments, the expanded GAA triplet-repeat sequence was found to be a source of position effect variegation (PEV) i.e., a source of heterochromatin spreading into adjacent euchromatin [5]. Consistent with this observation, evidence of heterochromatin formation was found in the immediate vicinity of the expanded GAA triplet-repeat in cells from FRDA patients [6]–[8]. More importantly, histone deacetylase (HDAC) inhibitors resulted in partial reversal of the gene silencing in patient-derived cells [6], indicating that heterochromatin formation is an important underlying mechanism for the transcriptional deficiency in FRDA. However, heterochromatin formation has not been convincingly demonstrated in any region other than intron 1 of the *FXN* gene in cells of FRDA patients, and exactly how transcriptional silencing occurs is not fully understood.

Here, we report that FRDA patients have a severe depletion of the chromatin insulator protein CTCF (CCCTC-binding factor) in the 5′ untranslated sequence (5′UTR) of the *FXN* gene. We also found that CTCF depletion was associated with higher levels of an antisense transcript, and heterochromatin formation involving the critical +1 nucleosome in the vicinity of the transcription start site (TSS) of the *FXN* gene. Knockdown of CTCF reproduced the deficiency of *FXN* gene transcription, and higher levels of antisense transcription. Our data support the hypothesis that CTCF depletion in FRDA constitutes an epigenetic switch that results in heterochromatin formation and deficiency of *FXN* gene transcription.

## Results

### CTCF Is Depleted in the 5′UTR of the *FXN* Gene in FRDA

**Figure 1:**
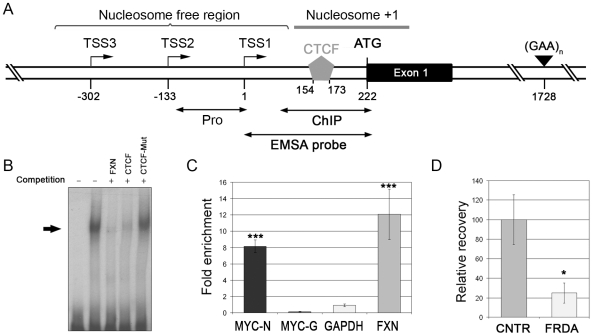
Depletion of CTCF in the *FXN* 5′UTR in FRDA. (**A**) Schematic representation of the relevant portion of the *FXN* gene showing the CTCF binding site in the 5′UTR, and the regions analyzed by EMSA and ChIP. All numbers are relative to TSS1. The region denoted as “Pro” is the portion of the promoter/5′UTR previously analyzed by other groups and shown not to have heterochromatin formation in FRDA [6], [8]. (**B**) EMSA with HeLa nuclear extract showing a single complex (arrow) that was competed with excess cold probe, CTCF binding oligonucleotide, but not with a mutant CTCF oligonucleotide, indicating that CTCF binds in the *FXN* 5′UTR. (**C**) ChIP with anti-CTCF in fibroblasts from non-FRDA controls showing enrichment of CTCF in the *FXN* 5′UTR *in vivo*. The CTCF insulator (MYC-N) and a non-binding site (MYC-G) in the *MYC* gene were used as positive and negative controls for CTCF enrichment *in vivo*. The enrichment at the *FXN* 5′UTR is comparable to the *MYC* locus. The GAPDH fragment used to normalize the ChIP data did not show CTCF enrichment. (**D**) ChIP with anti-CTCF in fibroblasts from two non-FRDA control fibroblasts (CNTR) and two FRDA patients showed significantly less occupancy of CTCF in FRDA. The data are from two independent chromatin preparations, with each experiment done in triplicate. Error bars = s.e.m.; “*” = *P*<0.05; “***” = *P*<0.001.

Publicly available ChIP-on-CHIP data [9] revealed a single CTCF binding site in the *FXN* gene that maps in the 5′UTR (+154 to +173, relative to the most proximal TSS [TSS1]) (Fig. 1A). Electrophoretic mobility shift assay (EMSA) using the 5′UTR as a probe and HeLa nuclear extract showed a single shifted complex (Fig. 1B). This complex was competed away with an oligonucleotide probe containing the consensus CTCF-binding site, but not with a control probe containing a mutant CTCF-binding sequence (Fig. 1B), indicating that the shift was due to CTCF binding in the 5′UTR. Chromatin immunoprecipitation (ChIP) with anti-CTCF using fibroblast cell lines from two normal individuals showed considerable enrichment of CTCF in the 5′UTR of the *FXN* gene *in vivo*, which was comparable to that seen at the *MYC* locus, used as a positive control (Fig. 1C). Strikingly, the same ChIP assay performed with fibroblast cell lines from two FRDA patients, who were homozygous for expanded GAA triplet-repeat sequences in intron 1 of the *FXN* gene, showed four-fold reduced occupancy of CTCF in the 5′UTR (Fig. 1D). These data indicate that CTCF is depleted from the 5′UTR of the *FXN* gene in FRDA patients. Comparison of CTCF occupancy at the *MYC* locus, and at another CTCF binding site at the *APBA1* (Amyloid beta A4 precursor protein-binding family A member 1) locus on chromosome 9q, in FRDA versus normal cell lines did not show a similar reduction, indicating that FRDA cells do not have a generalized defect of CTCF binding (Fig. S1). DNA methylation is known to interfere with CTCF binding [10], and there are two CpG dinucleotides associated with the CTCF binding site in the *FXN* 5′UTR. Given that FRDA patients have increased CpG methylation in intron 1 of the *FXN* gene [7], [8], we investigated if altered methylation in the 5′UTR was a possible mechanism for the depletion of CTCF. Bisulfite treatment, followed by sequencing of twelve individual clones from each of two FRDA and two control fibroblasts, did not show any alteration in methylation status at any of the nine CpG dinucleotides spanning the 5′UTR (including the two that overlap with the CTCF binding site), or in the first six CpG sites of exon 1. Furthermore, EMSA performed with HeLa nuclear extract and an *in vitro* methylated 5′UTR probe (methylation status of which was confirmed by bisulfite sequencing) showed the same major complex obtained with the unmethylated probe, which was competed away by excess cold unmethylated probe (Fig. S2), indicating that altered DNA methylation is unlikely to be the reason for CTCF depletion in the *FXN* 5′UTR in FRDA.

### Heterochromatin Formation Involving the +1 Nucleosome of the *FXN* Gene in FRDA

**Figure 2:**
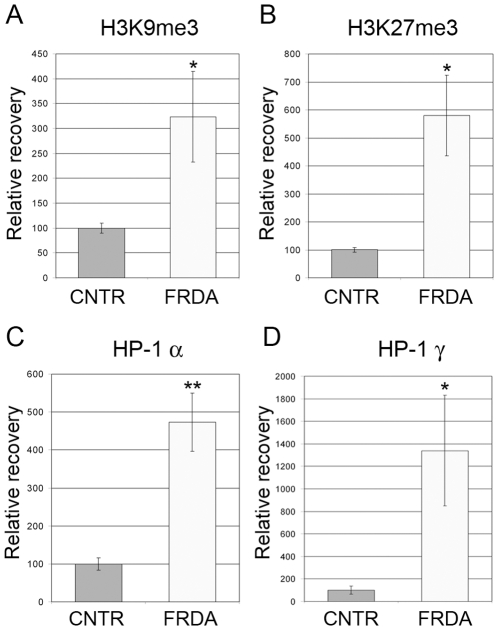
Heterochromatin formation in the *FXN* 5′UTR in FRDA patients. ChIP assays showing enrichment of (**A**) H3K9me3, (**B**) H3K27me3, and (**C,D**) heterochromatin protein 1 subunits HP-1α and HP-1γ, specifically in FRDA fibroblast cell lines versus non-FRDA controls (CNTR). All bars represent cumulative data from two fibroblast cell lines (FRDA or non-FRDA control), ChIP performed in triplicate, on two independent chromatin preparations. Error bars = s.e.m.; “*” = *P*<0.05; “**” = *P*<0.01.

We hypothesized that in FRDA patients depletion of CTCF, a known chromatin insulator protein [10], would be associated with heterochromatin formation in the 5′UTR. Publicly available, genome-wide, nucleosome occupancy data (nucleosome occupancy super-track; UCSC genome browser [11]–[13]) showed that the +1 nucleosome of the *FXN* gene encompassed the CTCF binding site in the 5′UTR, which was preceded by a long nucleosome free region containing the three reported TSS's of the *FXN* gene (denoted as TSS1-3 in Fig. 1A). ChIP followed by quantitative PCR of the region spanning the +1 nucleosome showed significant enrichment of histone modifications characteristic of heterochromatin formation specifically in FRDA cell lines. We detected significant enrichment of H3K9me3 and H3K27me3 (Fig. 2A,B). H3K9me3 is known to recruit heterochromatin protein 1 (HP1) via interaction with the latter's chromodomain. Indeed, we also detected significant enrichment of HP1 (both α and γ subunits were tested and found to be enriched) in FRDA compared with normal fibroblasts (Fig. 2C,D). These data indicate that CTCF depletion in FRDA patients is associated with heterochromatin formation involving the +1 nucleosome of the *FXN* gene.

### FRDA Patients Have Higher Levels of FAST-1

**Figure 3:**
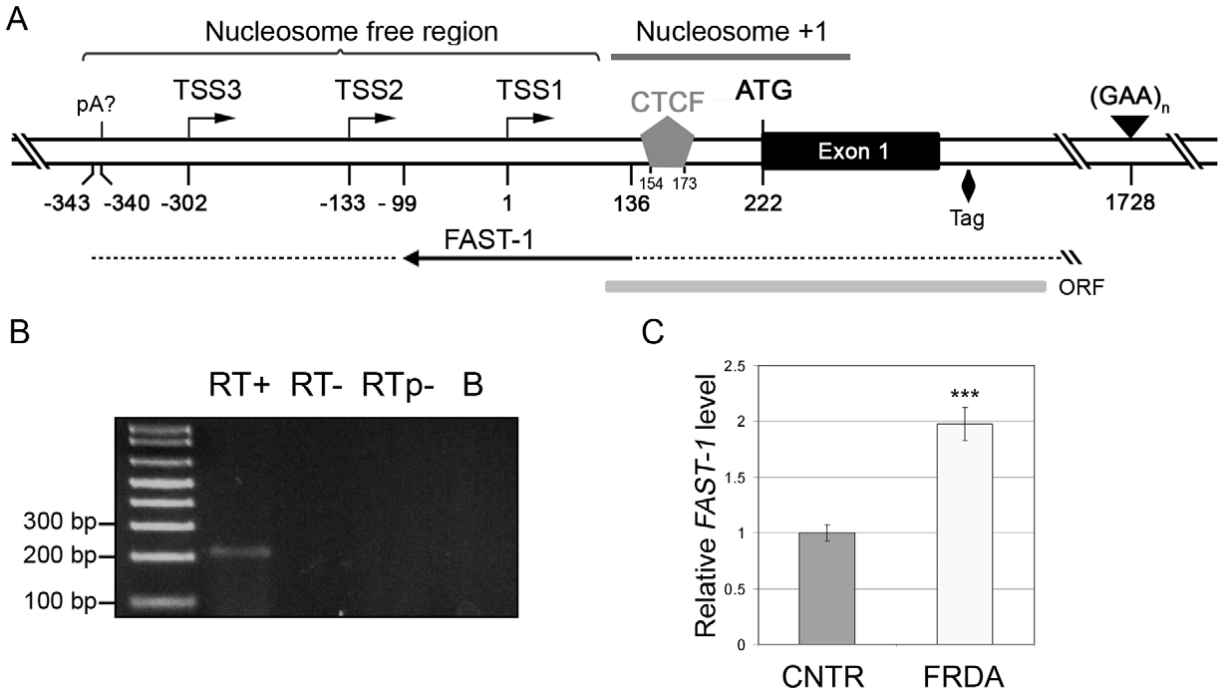
FAST-1, an antisense transcript overlapping with the CTCF binding site, is present at higher levels in FRDA patients. (**A, B**) Strand-specific RT-PCR showed the presence of an antisense transcript that overlaps with the *FXN* gene. The transcript extends at least from *FXN* intron 1 to upstream of the *FXN* TSS. The solid line represents the part of the transcript (207-bp product in **B**) that we could amplify using one round of PCR (with FAST-F1 and FAST-R1; see Methods), and the dotted line denotes the extended transcript that we detected via nested PCR. The FAST-1 product was not detected in a no-RT control (RT-), or when the RT reaction was performed without any exogenous primers (RTp-). The diamond symbol represents a sequence tag corresponding to FAST-1, identified in the antisense transcriptome [11]. The gray bar represents the extent of an open reading frame, and “pA” denotes a putative polyadenylation (pA) signal for FAST-1. All numbers are relative to TSS1. (**C**) Real-time RT-PCR (with FAST-F1 and FAST-R1) showed twice as much FAST-1 levels in FRDA cell lines versus non-FRDA controls (CNTR). The bars represent cumulative data from two fibroblast cell lines (FRDA or CNTR), RT-PCR in triplicate, from two independent experiments. Error bars = s.e.m.; “***” = *P*<0.001.

As a potential mediator of heterochromatin formation, we searched for an antisense transcript overlapping with the 5′UTR of the *FXN* gene. Strand-specific RT-PCR was carried out with a primer located upstream of *FXN* TSS3 (Fig. 3A) and RNA from normal fibroblasts, which revealed an antisense transcript that overlapped with the CTCF binding site (Fig. 3A,B). We have named this novel transcript FAST-1 (*FXN* Antisense Transcript – 1). No such product was detected in a “no-RT” reaction or in a “RT” reaction without exogenous primers (Fig. 3B), indicating that this product was not amplified from contaminating DNA or via spurious endogenous priming. Sequencing of the isolated transcript showed that it was a perfect match to the antisense strand of *FXN*, and BLAST analysis of this sequence showed only a single hit (to the *FXN* locus), indicating that FAST-1 originates from the *FXN* locus. RT-PCR experiments indicated that FAST-1 overlaps with the *FXN* gene, including at least portions of intron 1, exon 1, the 5′UTR, and extends further upstream into the nucleosome free region and upstream of *FXN* TSS3 (until -343; Fig. 3A). It is presently unclear if the putative polyadenylation sequence located upstream of *FXN* TSS3 (−340; Fig. 3A) is utilized by FAST-1. A tag corresponding to FAST-1 was identified in a recent study of the human antisense transcriptome (position 70840691 on the “-” strand of chromosome 9 in the HCT116 cell line) [14] (Papadopoulos, *personal communication*), which maps close to the start of intron 1 of the *FXN* gene (Fig. 3A), and further substantiates the existence of FAST-1. FAST-1 contains an open reading frame which encodes a putative peptide of 154 amino acids (70840410 to 70840874 on the “-” strand of chromosome 9, which spans the antisense sequence tag isolated in [14]), with homology to two uncharacterized open reading frames in rice. The open reading frame extends from *FXN* intron 1, through exon 1, and ends in the 5′UTR, and includes the CTCF binding site (Fig. 3A). However, the importance of this open reading frame is unclear since it is intact in human and chimpanzee, but contains premature stop codons in orangutan, rhesus, and marmoset, and the translational initiation site does not show a Kozak consensus sequence.

We hypothesized that FRDA patients would have higher levels of FAST-1, corresponding to the depletion of CTCF and heterochromatin formation in the *FXN* 5′UTR. Indeed, real-time RT-PCR to measure FAST-1 levels in FRDA versus normal fibroblasts revealed that patients had twice as much of this antisense transcript (Fig. 3C). It should be noted that our data are consistent with either actual overexpression of FAST-1, increased spreading of FAST-1 upstream beyond the CTCF binding site, or both.

### CTCF Knockdown Reproduces the Deficiency of *FXN* Transcript and Higher Levels of FAST-1 Seen in FRDA Cells

**Figure 4:**
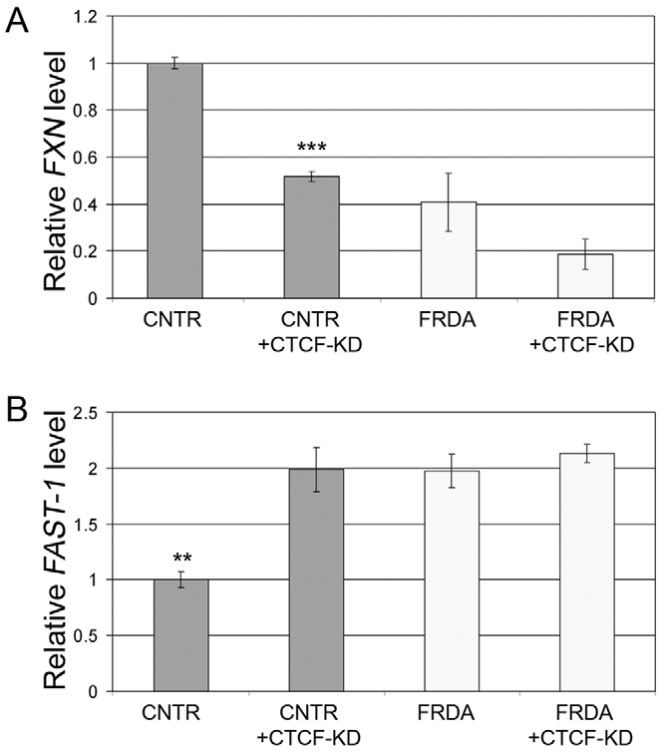
siRNA knockdown of CTCF in normal cells reproduces the deficiency of *FXN* transcript and higher levels of FAST-1 seen in FRDA cells. All bars represent cumulative data from two fibroblast cell lines (FRDA or CNTR), RT-PCR done in triplicate, from two independent experiments. Error bars = s.e.m.; “**” = *P*<0.01; “***” = *P*<0.001. (**A**) Real-time RT-PCR showed that knockdown of CTCF in normal fibroblasts (CNTR) resulted in significant reduction in levels of *FXN* transcript, which was similar to that seen in FRDA fibroblasts (the levels of *FXN* transcript in FRDA cells is similar to those reported previously [6], [17]). Knockdown of CTCF in FRDA cell lines seemed to further reduce *FXN* transcript levels but this was not statistically significant. (**B**) Real-time RT-PCR showed that knockdown of CTCF in normal fibroblasts (CNTR) resulted in significant increase in levels of FAST-1, which was similar to that seen in FRDA fibroblasts. Knockdown of CTCF in FRDA cells did not further increase FAST-1 levels, which remained around twice as high as in normal cells.

In order to test if CTCF depletion is responsible for the deficiency of *FXN* transcript and higher levels of FAST-1, as is seen in FRDA patients, siRNA-mediated knockdown of CTCF was performed in fibroblasts. Western blot analysis and quantitative RT-PCR confirmed the global knockdown of CTCF (Fig. S3, S4), and a micro-ChIP assay [15] was used to confirm depletion of CTCF specifically in the 5′UTR of the *FXN* gene (Fig. S5). Real-time RT-PCR showed a significant reduction in the levels of *FXN* transcript in siRNA treated fibroblasts from normal individuals, but no further reduction was seen in FRDA cells (Fig. 4A). This effect was unrelated to the transfection protocol *per se*, since no such effect was seen with a scrambled sequence control siRNA (Fig. S3, S4, S5, S6). Furthermore, real-time RT-PCR showed a corresponding doubling of the levels of FAST-1 upon siRNA knockdown in normal fibroblasts (Fig. 4B). Indeed, FAST-1 levels in normal fibroblasts were equalized to the level seen in FRDA cells via knockdown of CTCF. CTCF knockdown in FRDA cells did not result in further increase in FAST-1 levels. These data indicate that depletion of CTCF in normal cells is capable of reproducing the reciprocal deficiency of *FXN* transcript and higher levels of FAST-1, and is comparable in scale to what is seen naturally in FRDA cells.

## Discussion

The expanded GAA triplet-repeat sequence results in deficiency of *FXN* transcript via at least two mechanisms: deficient transcriptional elongation through the expanded repeat tract [3], [16], [17] and epigenetic silencing [6]–[8]. Previous studies detected evidence of heterochromatin formation in the immediate vicinity of the expanded GAA triplet-repeat in intron 1 in FRDA [6]–[8], however, it has remained unclear how this results in transcriptional silencing of the *FXN* gene. Our data are consistent with the model that CTCF depletion in the 5′UTR of the *FXN* gene in FRDA patients results in reduced *FXN* transcription via heterochromatin formation involving the critical +1 nucleosome, which offers a plausible mechanism for transcriptional silencing of the *FXN* gene, and is consistent with the observed transcriptional reactivation via administration of HDAC inhibitors [6]. It is interesting to note that previous unsuccessful attempts to detect heterochromatin formation in the upstream regions of the *FXN* gene in FRDA were directed to the nucleosome free region immediately upstream of TSS1 [6], [8]; the sequence we have analyzed here is ∼100 nucleotides downstream of this sequence, and represents the +1 nucleosome (Fig. 1A). The +1 nucleosome is an important regulator of transcription [18] and alteration of its chromatin organization in FRDA has the possibility of influencing *FXN* transcriptional initiation and/or elongation. It is also interesting to note that heterochromatin formation in the vicinity of the expanded GAA triplet-repeat in intron 1 of FRDA patients is different from what we have observed in the 5′UTR. The former is associated with increased CpG methylation at specific sites in intron 1, whereas we did not detect any alterations in DNA methylation in the 5′UTR. Instead, heterochromatin formation in the 5′UTR seems to be based on H3K9me3-mediated recruitment of HP1.

**Figure 5:**
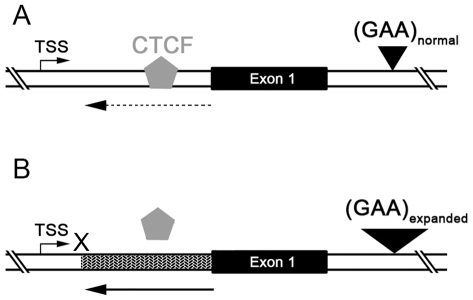
CTCF depletion, increased FAST-1 level, and heterochromatin formation in FRDA. (**A**) In non-FRDA cells there is low level of FAST-1 (dotted arrow), normal occupancy of CTCF and no heterochromatin formation in the 5′UTR. (**B**) Our data are compatible with the following model in FRDA cells: CTCF is depleted from the 5′UTR, followed by increased levels (or spreading) of FAST-1 into the 5′UTR (arrow), which induces H3K9me3 and HP-1 mediated heterochromatin formation (filled pattern), leading to transcriptional silencing of the *FXN* gene.

Antisense transcripts have been implicated in heterochromatin formation via H3K9me3 and subsequent recruitment of HP1 [19]–[21]. Most human genes are known to have transcripts that emanate from, and head upstream of, their transcription start sites [22]–[24]. However, these are technically not “antisense” transcripts since they do not overlap with sense transcripts. Nevertheless, ∼15% of genes are known to have *bona fide* antisense transcripts [14], [25] that may serve to regulate the expression of their corresponding sense transcripts. The discovery of FAST-1 is therefore not surprising in itself, however, the detection of higher levels of FAST-1 in the region showing heterochromatin formation in FRDA patients offers a plausible mechanistic basis for the epigenetic abnormality involving the +1 nucleosome [19]–[21]. Moreover, since knockdown of CTCF in normal fibroblasts reproduced the increase in levels of FAST-1 seen in FRDA fibroblasts, it suggests that CTCF depletion is required for increased FAST-1 levels or spreading. Indeed, increased spreading, or level of expression, of antisense transcripts has been shown to induce epigenetic silencing of the corresponding “sense” gene. A genomic deletion involving an intervening transcriptional termination signal resulted in abnormal spreading of an antisense transcript into the human α-globin gene and resulted in transcriptional silencing associated with DNA hypermethylation [26]. Furthermore, the microRNA-mediated transcriptional silencing via heterochromatin formation, which is well characterized in *S. pombe*, is also conserved in mammalian cells [27], and most likely results from targeting of the sense transcript, rather than the genomic DNA itself [28]. Indeed, Yu et al. [29] directly demonstrated that mammalian cells are capable of establishing heterochromatin formation via overexpression of an antisense transcript. Therefore, taken together, our data support the model that CTCF depletion constitutes an epigenetic switch in FRDA that allows increased expression or spreading of FAST-1, heterochromatin formation involving the +1 nucleosome, and transcriptional silencing of the *FXN* gene (Fig. 5).

CTCF, a multi zinc-finger protein, binds ∼15,000 sites in the human genome [9]. Indeed, the site we identified in the *FXN* 5′UTR is one of ∼6,000 sites that are located in the vicinity of transcription start sites of human genes. CTCF is a chromatin insulator that acts by establishment of chromatin topological domains via CTCF-CTCF interactions, and tethering to the nucleolar surface via CTCF-nucleophosmin interactions. This higher-order chromatin organization is known to regulate gene expression via creation of boundaries in chromatin [30]–[32] such that they prevent (or facilitate) interactions between enhancers and promoter sequences (enhancer-blocking insulators), or by preventing the spread of heterochromatin (e.g. via PEV) by creating a barrier between active and silenced chromatin (barrier insulators). This higher-order organization afforded by chromatin insulators is an important regulator of normal gene expression [30]–[32]. Our data suggest various potential (non mutually-exclusive) ways by which CTCF depletion could result in heterochromatin formation and deficiency of *FXN* transcript. It is possible that a CTCF-mediated, enhancer-blocking insulator normally keeps FAST-1 expression low. Local depletion of CTCF in FRDA could disrupt this enhancer-blocking activity, allowing FAST-1 overexpression (as occurs in the paternal *IGF2* allele at the imprinted *H19-IGF2* locus). Another possibility is that CTCF depletion in the *FXN* 5′UTR could allow increased spreading of FAST-1 to upstream sequences. FAST-1 overexpression or increased spreading could result in heterochromatin formation and *FXN* gene silencing via H3K9me3 and recruitment of HP-1 [19]–[21]. Indeed, CTCF is known to limit the spread of an antisense transcript at the human *DMPK* locus [33]. CTCF is displaced in the presence of very large expansions of a nearby CTG triplet-repeat sequence, resulting in spreading of antisense transcription and heterochromatin formation beyond their limited confines, leading to congenital myotonic dystrophy [33]. Another possibility is that the depletion of CTCF in the 5′UTR could disrupt a CTCF-mediated topological segregation of the expanded GAA triplet-repeat, a known source of PEV [5], from the 5′UTR and TSS of the *FXN* gene. This would allow the upstream spreading of heterochromatin from the expanded GAA triplet-repeat in FRDA patients, thus resulting in *FXN* transcriptional deficiency.

It remains unclear as to how the expanded GAA triplet-repeat, the cause of FRDA [2], results in displacement of CTCF. Cohesin, which is essential for sister chromatid cohesion [34], often colocalizes with CTCF [35]–[37]. Cohesin also cooperates with CTCF in regulating gene expression [38], and is required for CTCF binding at some sites [35], [36]. An intriguing possibility is that the expanded GAA triplet-repeat sequence, possibly via formation of a non-B DNA structure [3], [16], [39], may compromise the loading, binding, or stability of cohesin and thereby promote CTCF displacement. Another possibility, which cannot be ruled out presently, is that FAST-1 overexpression may itself displace CTCF from the 5′UTR, either directly or secondary to +1 nucleosomal modification and/or displacement. Indeed, lipopolysaccharide-induced overexpression of the chicken lysozyme gene is mediated by increased antisense transcription that results in repositioning of a nucleosome over the CTCF binding site leading to its eviction [40]. The coincidence of the CTCF binding site in the *FXN* 5′UTR and the +1 nucleosome tends to support this type of mechanism, although our data did demonstrate that FAST-1 overexpression occurred as a result of CTCF deficiency.

The discovery that HDAC inhibitors result in reversal of the *FXN* transcriptional silencing has led to their use in currently ongoing clinical trials. Our findings provide a mechanistic basis for the transcriptional silencing of the *FXN* gene in Friedreich ataxia that is consistent with this response to HDAC inhibitors, and elucidate a rational basis for their action. Moreover, the identification of localized CTCF depletion and increased FAST-1 levels offer potential therapeutic targets for the specific reactivation of the transcriptionally silenced *FXN* gene in FRDA.

## Materials and Methods

### Cell Lines

Fibroblasts from two healthy subjects (GM04503, GM07492) and two FRDA patients (GM03665, GM04078) were obtained from Coriell Cell Repository. Expanded GAA alleles for the two FRDA patients were 445 & 740 triplets (GM03665), and 345 & 470 triplets (GM04078). All experiments were performed with early passages of the four cell lines.

### Electrophoretic Mobility Shift Assay

A 231-bp PCR fragment (position −222 to +9 relative to the initiation codon) containing the putative CTCF binding site in the 5′UTR of the *FXN* gene was used as the probe. The end-labeled probe was incubated with HeLa nuclear extract (5 µg; Millipore) for 40 minutes at room temperature in binding buffer (10 mM Tris, pH 7.5, 50 mM NaCl, 1 mM dithiothreitol, 1 mM EDTA, 5% glycerol, and poly dI-dC), and resolved on a 5% native polyacrylamide gel. Cold competition was performed using excess unlabeled probe, a CTCF consensus oligonucleotide (sc-2613; Santa Cruz), or a mutant CTCF oligonucleotide (sc-2614; Santa Cruz). Methylated probe for EMSA was generated via *in vitro* methylation using M.SssI CpG methyltransferase and S-adenosylmethionine (New England BioLabs).

### Bisulfite Sequencing for Detection of CpG Methylation

200 ng of genomic DNA was treated using the EZ DNA Methylation-Direct Kit (Zymo Research) to convert unmethylated cytosines to uracil. PCR was performed using the following primer pairs: CTCF-meth-F1 5′-GCTCCGTCTAGATTGTGTATGTATTTATTTTTTAG-3′ and CTCF-meth-R1 5′-GATGCGCTGCAGAAAATAAAACCAACTCTACC-3′ followed by nested PCR with primers: CTCF-meth-F2 5′-GCTCCGTCTAGAGGTTGTATTTCGTGTTTTGT-3′ and CTCF-meth-R2 5′-GATGCGCTGCAGCTAAACCTAAACTAAACTAAATAAC-3′. The XbaI and PstI sites in the forward and reverse primers, respectively, were used to clone the PCR products into pUC19. Twelve clones were sequenced for each DNA sample. Cytosine to uracil conversion was complete at all non CpG sites.

### Chromatin Immunoprecipitation (ChIP)

We used the ChIP-IT Express kit (Active Motif). Immunoprecipitations were performed overnight at 4°C using anti-CTCF (Millipore 07-729), anti-H3K9me3 (Millipore 17-625), anti-H3K27me3 (Millipore 07-449), anti-HP1α (Millipore 07-346), or anti-HP1γ (Millipore 07-332). The immunoprecipitated DNA and the input DNA were analyzed by real-time PCR using the ΔΔCt method on the Eppendorf RealPlex-4 Mastercycler with Power SYBR Green PCR Mastermix (Applied Biosystems) and the following primers:

FXN-ChIP-forward 5′-TCCTGAGGTCTAACCTCTAGCTGC-3′ and

FXN-ChIP-reverse 5′-CGAGAGTCCACATGCTGCTCC-3′

GAPDH-ChIP-forward 5′-CCTCCCGCTTCGCTCTCT-3′ and

GAPDH-ChIP-reverse 5′-GGTTTCTCTCCGCCCGTCT-3′

Each value of immunoprecipitated DNA, normalized to GAPDH, was further normalized to the corresponding input DNA. All ChIP experiments, which were performed in triplicate, were done using two independent chromatin preparations. All data are from the two FRDA cell lines combined versus the two normal controls combined. The human CTCF insulator (MYC-N) and a non-binding site (MYC-G) in the *MYC* gene were used as positive and negative controls for CTCF binding [41]. The region of GAPDH used to normalize the ChIP data was confirmed to not enrich CTCF by comparing it with another region of the GAPDH gene located far away from any known CTCF binding sites (using the following primers: 5′-TACTAGCGGTTTTACGGGCG-3′ and 5′-TCGAACAGGAGGAGCAGAGAGCGA-3′). CTCF enrichment at the *APBA1* locus on chromosome 9 was tested with primers: 5′-CCTCAATCGCCTGGGAATCT-3′ and 5′-GCCCTCCCCAGCAGACA-3′. Micro-ChIP was used to test the depletion of CTCF specifically in the *FXN* gene. This was essentially performed as previously described [15]. The only modifications we implemented included the use of 25,000 cells and optimization of the sonication step to yield a modal sheared chromatin size of ∼500 bp.

### Quantitative RT-PCR

Total RNA was isolated with TriPure (Roche), and cDNA was synthesized using the QuantiTect reverse transcription kit (Qiagen). Relative transcript levels were measured by real-time PCR as above, and normalized to HPRT. *FXN* transcript levels were detected as previously described [6]. Detection of FAST-1 was accomplished by reverse transcription using a strand-specific primer (FAST-RT: 5′- CCAAGCAGCCTCAATTTGTG-3′). PCR was performed with FAST-F1 (5′-GTGGGGGAGCAGCTAGAGG-3′) and FAST-R1 (5′-CACTTCCCAGCAAGACAGC-3′) (note: “F” and “R” designations are in the FAST-1 orientation). PCR for FAST-1 was performed using either Taq DNA polymerase (Roche) or the Power SYBR Green PCR Mastermix (Applied Biosystems), and 35 cycles of 30 s at 95°C, 30 s at 62°C, and 30 s at 70°C. Relative FAST-1 levels were quantified by real-time PCR (as above), and expression levels were normalized to HPRT. Statistical analysis was performed with triplicate data from two independent experiments.

### siRNA Knockdown of CTCF

Stealth RNAi for CTCF (Invitrogen; 6 µg) was used to transfect cells in 6-well plates using Lipofectamine 2000 (Invitrogen). Stealth RNAi Negative Universal Control (Invitrogen) was used as the scrambled sequence control. Cells were harvested 48 hr post-transfection. CTCF knockdown was confirmed using quantitative RT-PCR (with primers described in [41]) and western blot analysis with anti-CTCF antibody (Millipore 07-729). β-actin (Abcam 8227) was used as the loading control for the western blot analysis.

## Supporting Information

Figure S1 FRDA cells do not have a generalized defect of CTCF binding. ChIP with anti-CTCF in fibroblasts from FRDA and non-FRDA controls (CNTR) showed no reduction in enrichment of CTCF at the MYC-N insulator and the *APBA1* (Amyloid beta A4 precursor protein-binding family A member 1) locus on chromosome 9q *in vivo*. Whereas the *APBA1* locus showed no difference, the MYC-N site showed a slight increase in FRDA cells, the biological significance of which is unclear.
(0.85 MB TIF)

Figure S2 CTCF binds *in vitro* to the methylated *FXN* 5UTR. EMSA performed with HeLa nuclear extract and an *in vitro* methylated 5′UTR probe (methylation status of which was confirmed by bisulfite sequencing) showed a similar major complex as with the unmethylated probe. This complex was competed away by excess cold unmethylated probe, indicating that CTCF binds the 5′UTR irrespective of methylation status and that altered DNA methylation is unlikely to be the reason for CTCF depletion in the *FXN* 5′UTR in FRDA.
(0.64 MB TIF)

Figure S3 Western blot analysis showing siRNA mediated knockdown of CTCF protein. Fibroblasts from non-FRDA controls, either untransfected (UT), or treated with scrambled control siRNA (SC) or with a specific CTCF siRNA are shown. Western blot analysis with anti-CTCF and anti β-actin antibodies showed reduction of CTCF protein specifically in cells treated with CTCF siRNA.
(0.19 MB TIF)

Figure S4 Quantitative RT-PCR analysis showing siRNA mediated knockdown of CTCF transcript. Fibroblasts from non-FRDA controls, either untransfected (UT), or treated with scrambled control siRNA (SC) or with a specific CTCF siRNA are shown. Quantitative RT-PCR analysis of relative CTCF transcript levels (normalized to HPRT) showed reduction of CTCF transcript specifically in cells treated with CTCF siRNA. Error bars = s.e.m.; “*” = P<0.05.
(0.57 MB TIF)

Figure S5 CTCF knockdown results in depletion of CTCF at the *FXN* locus. Micro-ChIP assay performed on non-FRDA fibroblasts, either untransfected (UT) or treated with scrambled control siRNA (SC) or specific CTCF siRNA showed reduced CTCF occupancy at the *FXN* locus specifically in cells treated with CTCF siRNA. Error bars = s.e.m.; “*” = P<0.05.
(0.57 MB TIF)

Figure S6 CTCF knockdown results in deficiency of *FXN* transcript. Quantitative RT-PCR performed on non-FRDA fibroblasts, either untransfected (UT) or treated with scrambled control siRNA (SC) or specific CTCF siRNA showed reduced levels of *FXN* transcript (normalized to HPRT) specifically in cells treated with CTCF siRNA. Error bars = s.e.m.; “*” = P<0.05.
(0.57 MB TIF)
