# Supplementary figures and images for: Epigenetic Silencing in Friedreich Ataxia Is Associated with Depletion of CTCF (CCCTC-Binding Factor) and Antisense Transcription
Source: PLoS One. 2009 Nov 19;4(11):e7914. doi: 10.1371/journal.pone.0007914 (PMC2780319; doi:10.1371/journal.pone.0007914)

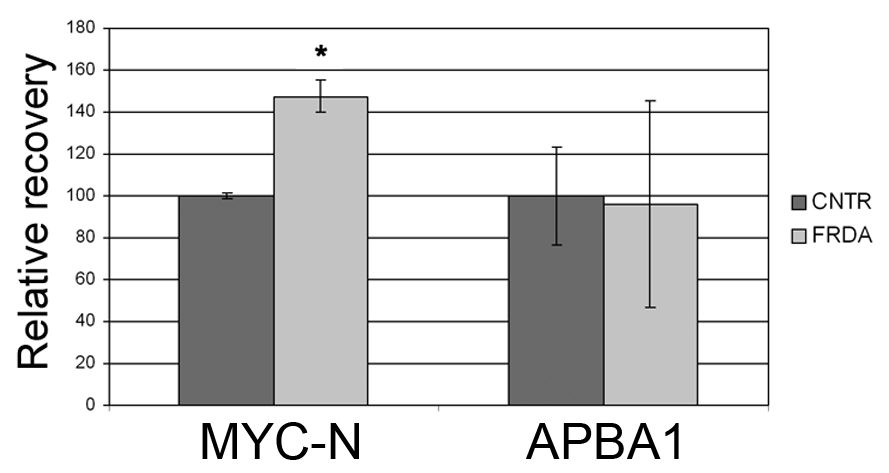

Supplement: Figure S1 — FRDA cells do not have a generalized defect of CTCF binding. ChIP with anti-CTCF in fibroblasts from FRDA and non-FRDA controls (CNTR) showed no reduction in enrichment of CTCF at the MYC-N insulator and the APBA1 (Amyloid beta A4 precursor protein-binding family A member 1) locus on chromosome 9q in vivo. Whereas the APBA1 locus showed no difference, the MYC-N site showed a slight increase in FRDA cells, the biological significance of which is unclear. (0.85 MB TIF) [file pone.0007914.s001.tif]

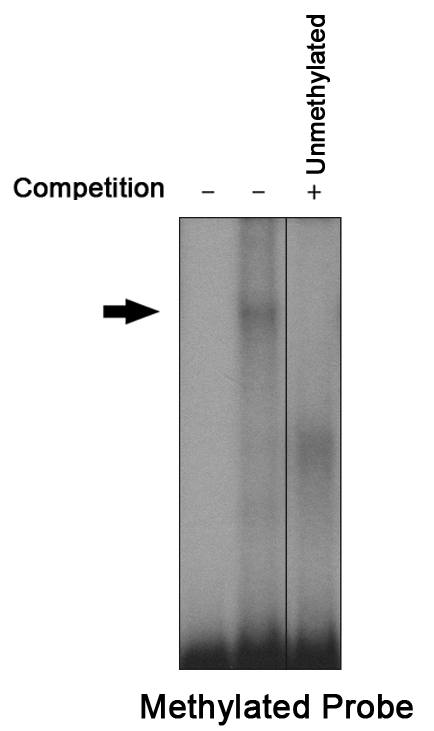

Supplement: Figure S2 — CTCF binds in vitro to the methylated FXN 5UTR. EMSA performed with HeLa nuclear extract and an in vitro methylated 5′UTR probe (methylation status of which was confirmed by bisulfite sequencing) showed a similar major complex as with the unmethylated probe. This complex was competed away by excess cold unmethylated probe, indicating that CTCF binds the 5′UTR irrespective of methylation status and that altered DNA methylation is unlikely to be the reason for CTCF depletion in the FXN 5′UTR in FRDA. (0.64 MB TIF) [file pone.0007914.s002.tif]

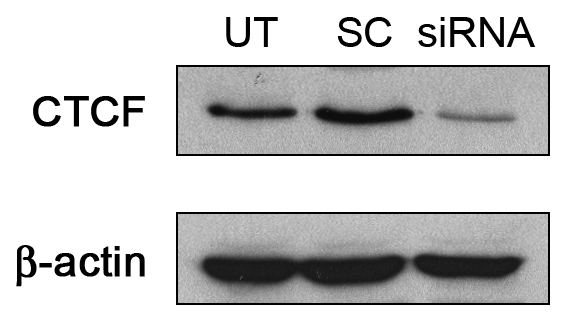

Supplement: Figure S3 — Western blot analysis showing siRNA mediated knockdown of CTCF protein. Fibroblasts from non-FRDA controls, either untransfected (UT), or treated with scrambled control siRNA (SC) or with a specific CTCF siRNA are shown. Western blot analysis with anti-CTCF and anti β-actin antibodies showed reduction of CTCF protein specifically in cells treated with CTCF siRNA. (0.19 MB TIF) [file pone.0007914.s003.tif]

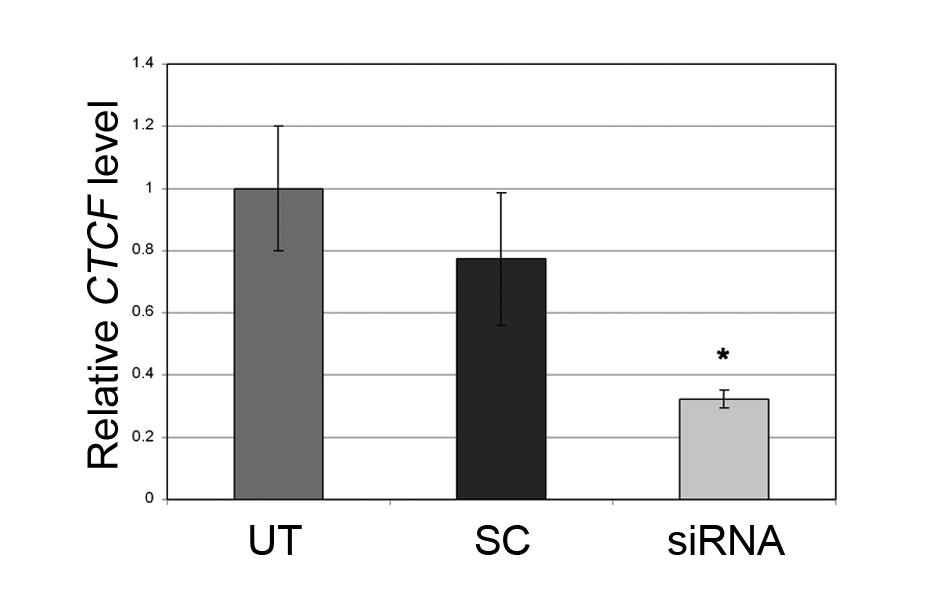

Supplement: Figure S4 — Quantitative RT-PCR analysis showing siRNA mediated knockdown of CTCF transcript. Fibroblasts from non-FRDA controls, either untransfected (UT), or treated with scrambled control siRNA (SC) or with a specific CTCF siRNA are shown. Quantitative RT-PCR analysis of relative CTCF transcript levels (normalized to HPRT) showed reduction of CTCF transcript specifically in cells treated with CTCF siRNA. Error bars = s.e.m.; “*” = P<0.05. (0.57 MB TIF) [file pone.0007914.s004.tif]

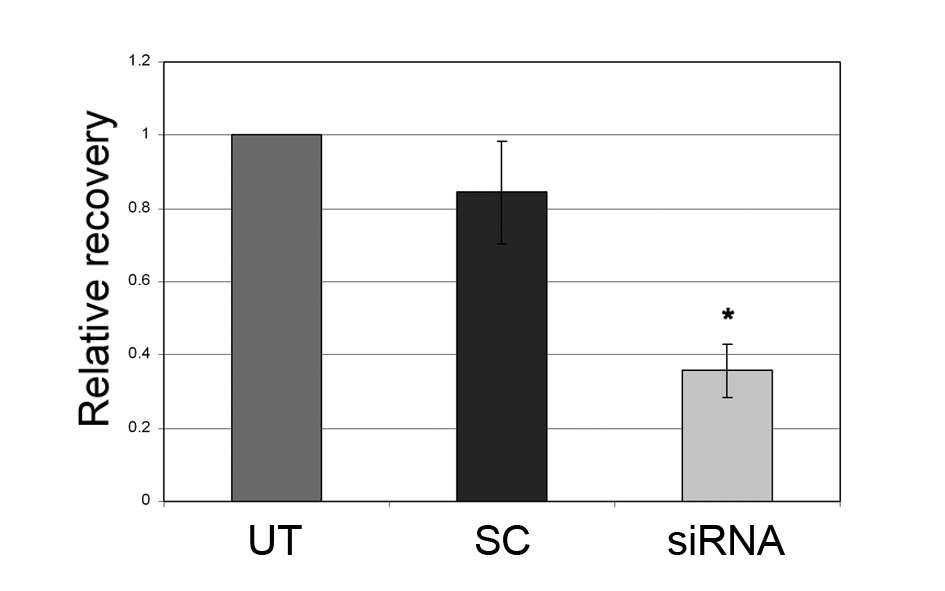

Supplement: Figure S5 — CTCF knockdown results in depletion of CTCF at the FXN locus. Micro-ChIP assay performed on non-FRDA fibroblasts, either untransfected (UT) or treated with scrambled control siRNA (SC) or specific CTCF siRNA showed reduced CTCF occupancy at the FXN locus specifically in cells treated with CTCF siRNA. Error bars = s.e.m.; “*” = P<0.05. (0.57 MB TIF) [file pone.0007914.s005.tif]

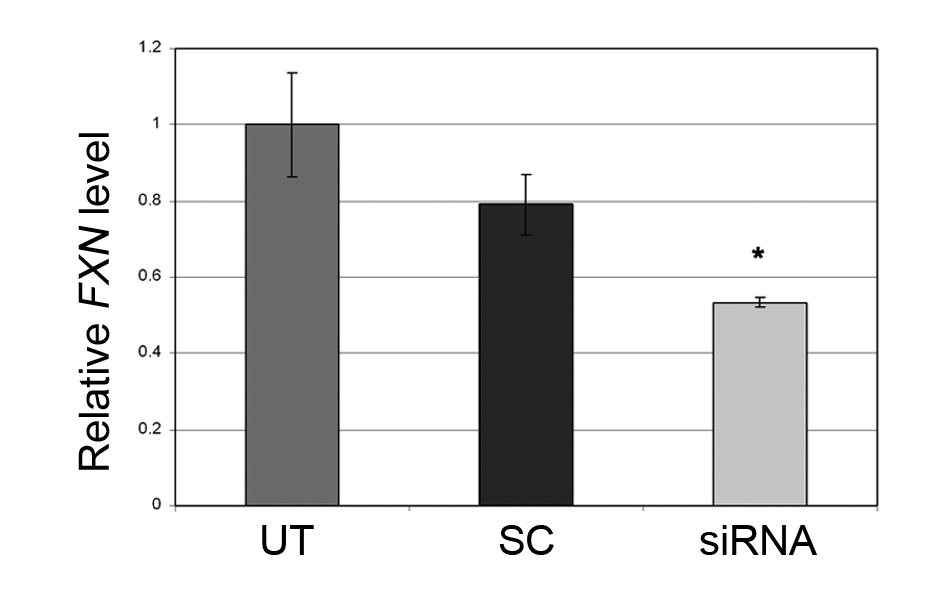

Supplement: Figure S6 — CTCF knockdown results in deficiency of FXN transcript. Quantitative RT-PCR performed on non-FRDA fibroblasts, either untransfected (UT) or treated with scrambled control siRNA (SC) or specific CTCF siRNA showed reduced levels of FXN transcript (normalized to HPRT) specifically in cells treated with CTCF siRNA. Error bars = s.e.m.; “*” = P<0.05. (0.57 MB TIF) [file pone.0007914.s006.tif]
